# Continuous Production of Pure Titanium with Ultrafine to Nanocrystalline Microstructure

**DOI:** 10.3390/ma13020336

**Published:** 2020-01-11

**Authors:** Kateřina Mertová, Jan Palán, Michal Duchek, Tomáš Studecký, Jan Džugan, Ivana Poláková

**Affiliations:** COMTES FHT a.s., Prumyslova 995, 334 41 Dobrany, Czech Republic; jan.palan@outlook.com (J.P.); michal.duchek@comtesfht.cz (M.D.); tomas.studecky@comtesfht.cz (T.S.); jan.dzugan@comtesfht.cz (J.D.); ivana.polakova@comtesfht.cz (I.P.)

**Keywords:** continuous production, pure titanium, severe plastic deformation

## Abstract

This work deals with the application of the Conform SPD (Severe Plastic Deformation) continuous extrusion process for ultrafine to nanostructured pure titanium production. The process has been derived from the Equal Channel Angular Pressing (ECAP) technique but, unlike ECAP, it offers continuous production of high-strength wire. This study describes the Conform SPD process combined with subsequent cold working (rotary swaging technique), its potential for commercial application, and the properties of high-strength wires of pure titanium. High-strength wire of titanium Grade 4 is the product. Titanium Grade 4 reaches ultimate strengths up to 1320 MPa. This value is more than twice the ultimate strength of the unprocessed material. The typical grain size upon processing ranges from 200 to 500 nm. Process development supported by FEM analysis together with detailed microstructure characterization accompanied by mechanical properties investigation is presented.

## 1. Introduction

In recent years, a lot of attention has been paid to the study of the material phenomena called Severe Plastic Deformation (SPD) [1,2]. SPD is a general term describing a group of metalworking techniques imposing very large strains, typically involving a complex stress state or high shear, resulting in a high defect density and the creation of equiaxed “ultrafine” grains [1,3]. 

Commercially pure titanium with high mechanical properties becomes increasingly attractive for medical applications. A major factor in this development is the recent studies which confirmed the potential hazards posed by the most widely used titanium alloy, Ti6Al4V [4,5,6]. The alloy may release aluminium and vanadium ions into the human body. These elements are toxic for humans [4,7]. Aluminium may even cause Alzheimer’s disease [7]. Commercially pure titanium exhibits high biocompatibility [6], but yields low mechanical properties. Therefore, technology leading to enhancement of mechanical properties for pure titanium are being investigated. Among them, several deformation processes providing nanostructured materials seem, at present, to be very promising. The high strength of such material would enable implants to be smaller. With a smaller load-bearing cross-sectional area, less human tissue is intruded and the implants may be more suitable for children and animals [2].

**Figure 1:**
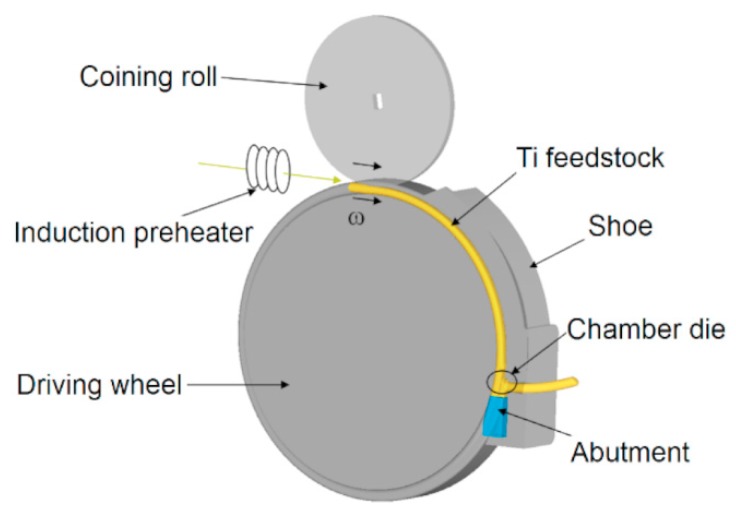
Schematic representation of the Conform SPD technique [24].

Today the most widely used SPD processes include HPT (High-Pressure Torsion), ARB (Accumulative Roll-Bonding) and ECAP (Equal-Channel Angular Pressing) [1]. However, these methods are not suitable for processing large volumes of metals, since they are established for small volume specimens and, moreover, are discontinuous [8,9]. Thus, recent research efforts have been focused on the development of continuous processes based, for example, on ECAP [10,11,12]. The original Conform SPD process is usually used for continuous extrusion of aluminium and copper profiles on an industrial scale. However, it delivers only a semi-product geometrical change without significant microstructural modification [12,13,14]. At COMTES FHT, the method has been modified in order to refine the structure during the forming process; and thus Conform SPD (Figure 1) was developed. This Conform SPD significantly improved the final mechanical properties [10,11,15]. The grain refinement is achieved thanks to the shear deformation and high hydrostatic pressure inside the processing chamber die which was designed based on experiences with the ECAP procedure [16,17]. SPD processes are usually applied in a multi-pass process, which means the grain structure is gradually refined with the increasing number of passes [18,19]. Another method is to combine the SPD process with cold working. It has already been proven that the combination of processes can lead to even higher mechanical properties than in the case of purely SPD processing [20,21,22,23]. 

This paper deals with the development of continuous technology for commercially pure titanium (titanium Grade 4) processing combining Conform SPD and rotary swaging leading to high-strength wire production. FEM analysis of the Conform SPD process is demonstrated here followed by a detailed study of the experimental material after processing with the use of optical and transmission electron microscopy and mechanical tests demonstrating the achieved material state.

## 2. Materials and Methods 

Commercially pure Grade 4 titanium (ASTM B348 Gr4) was processed. Its chemical composition is given in Table 1. The composition was measured by means of the Bruker Q4 Tasman optical emission spectrometer. The initial diameter of the feedstock was 10 mm.

The schematic picture of the SPD process is shown in Figure 1. Thanks to its axisymmetry, only one half of the equipment and feedstock were used for computation. In the deformation zone, titanium was considered to be plastic, whereas the other parts of the model were deemed rigid. The friction between the driving wheel and titanium bar plays a significant role in the mathematical model. The contact condition was set between forming wheel and feedstock. This condition ensured that the workpiece could not slip in the forming groove. The friction between the workpiece and the other tools was set to μ_f_ = 0.72. This value is recommended for hot forming by a software developer. These values were also verified by the pin on disc experiment. The temperature fields have only been studied in the titanium feedstock. The impact of the environment was neglected due to the rate of temperature changes. About 80,000 elements were created within the titanium feedstock. The models of the chamber die and its surroundings were fine-meshed. For more information of the FEM model, see [24]. Temperature-dependent material characteristics of titanium Grade 4 were obtained from JMatPro on the basis of the material’s chemical composition.

Two processes were considered: Conform SPD and rotary swaging of cold wire. All heating in the processes was generated by severe plastic deformation. During Conform SPD processing, the temperature of the die chamber was measured by a sheathed thermocouple. The feedstock orientation was identical in all passes and has a diameter of 10 millimetres. The output diameter of the wire was also 10 millimetres. The wheel speed was set to 0.5 rpm. This Conform SPD machine has a designed channel angle of 90°. Up to 4 passes Conform SPD processing were applied prior to subsequent processing of the experimental rods. Rotary swaging was performed on as-received wire and on a wire after one and three Conform SPD passes at ambient temperature. The objective was to analyse the impact of work hardening on ultrafine to nanocrystalline material. The stock was fed into the machine at a speed of approximately 0.5 m/s. In a single pass, the reduction of the cross-sectional area was 20%. The maximum final investigated reduction of the cross-sectional area was 90% which corresponds to a true strain of 2.2. 

Metallographic analysis of specimens was performed after grinding and subsequent polishing of the samples. The pictures of the microstructure were obtained by etching in Kroll reagent. An optical microscope Carl Zeiss-Observer.Z1m (Carl Zeiss AG, Oberkochen, Germany) in a mode of bright-field illumination was used for structure image capture.

For the purpose of observation in the transmission electron microscope (TEM), thin foils were prepared with final electrolytic thinning in a Tenupol 5 device, using a solution of 300 mL CH_3_OH + 175 mL 2-butanol + 30 mL HClO_4_ at a temperature of −10 °C and a voltage of 40 V. The TEM analysis was performed in a JEOL 200CX (JOEL, Tokyo, Japan) instrument with an acceleration voltage of 200 kV. Selective electron diffraction was used for phase identification. Grain size was measured using the linear intercept method. The sampling was carried out in the transverse direction.

Tensile testing was carried out at room temperature under quasi-static loading conditions with the use of an electromechanical testing machine. The average values from three valid measurement are plotted in the results section. The strain was measured with the use of a mechanical extensometer. The values of yield stress at 0.2% deformation (OYS), ultimate stress (UTS), elongation (A) and reduction of area (RA) were evaluated.

Microhardness was measured by means of a Durascan 50 automatic hardness tester. HV1 Vickers hardness values were measured in the transverse direction on samples after Conform SPD processing. The hardness was calculated from five valid measurements and the average value was calculated.

## 3. Results

### 3.1. Conform SPD Process Analysis 

**Figure 2:**
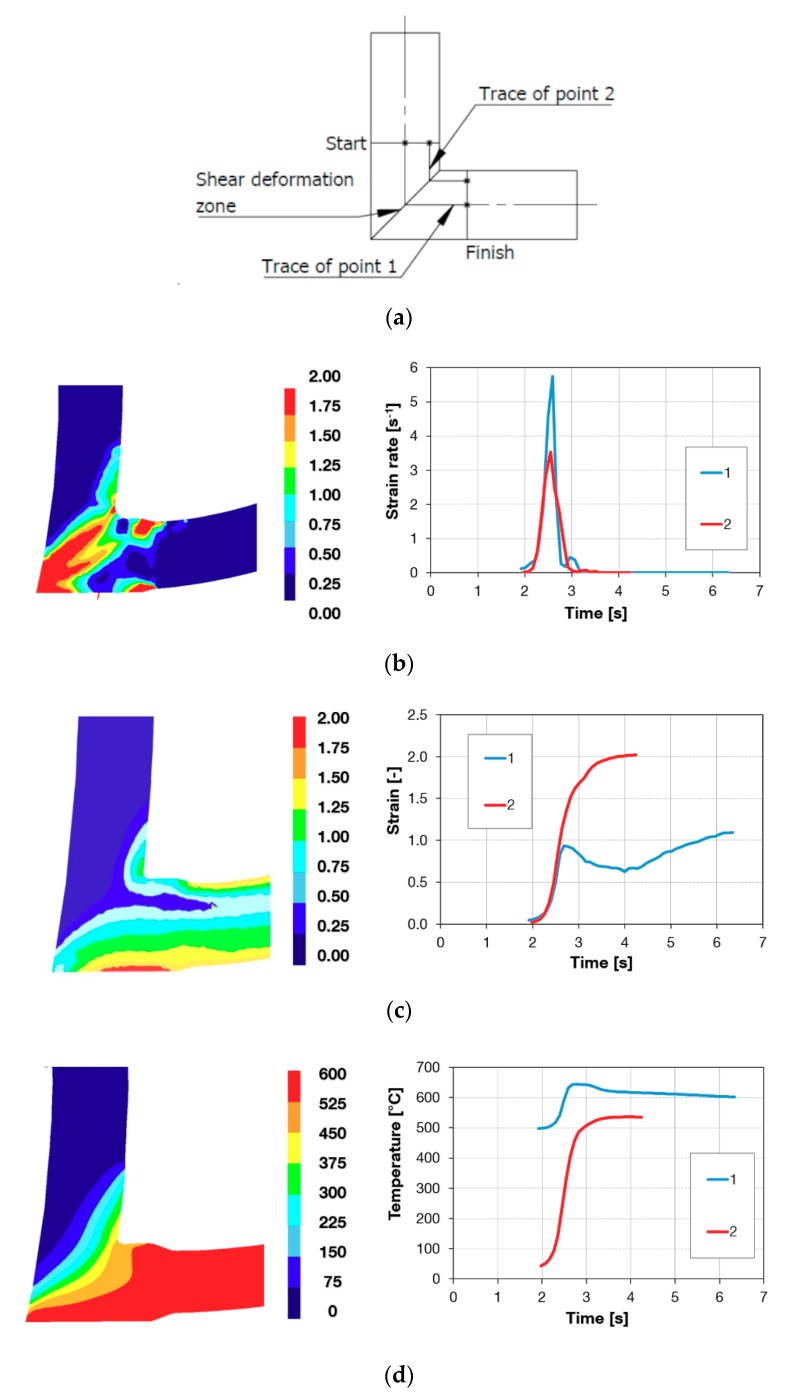
Result of FEM simulation: (**a**) Paths of the points under examination; (**b**) Strain rate distribution [s^−1^] and time trace of strain rate for points 1 and 2; (**c**) true strain distribution [-] and time relation of true strain for points 1 and 2; (**d**) temperature distribution over time for tracking points dependences of temperature for points 1 and 2.

The Conform SPD process was analysed using the FEM software DEFORM. Figure 2 shows the distributions of strain rate, true strain and temperature during the process, and the time dependences of these quantities for selected locations. These locations are identified as points in Figure 2a. Point 1 was in the centre of the blank and point 2 was located near the top of the chamber. At these points, time histories of strain rate, true strain and temperature were recorded. The recording interval included the shear deformation region between the Start and Finish line segments indicated in the schematic drawing. These three quantities are crucial to all SPD processes, as they have a significant impact on the resultant properties of the workpiece. Figure 2b shows the strain rate distribution, which is relatively non-uniform. The highest strain rate can be seen in the shear deformation zone on the left side of Figure 2b. The scale limit was modified for better differentiation. Strain rate is reduced at the top of the die chamber. This is shown by the related time history in Figure 2b on the right side of figure. The distribution of true strain is illustrated in Figure 2c. Higher strain is achieved near the surface of the workpiece due to friction. This can be seen in the time history in the same figure. At the end of the pass, point 2 shows higher strain. The temperature distribution in Figure 2d shows that severe deformation generates appreciable amounts of heat. The calculation suggests that the temperature reaches 600 °C after the shear deformation zone (see the left side of Figure 2d). The effect of the initial temperature of the blank on the final temperature was found to be negligible.

### 3.2. Metallographic Characterisation

**Figure 3:**
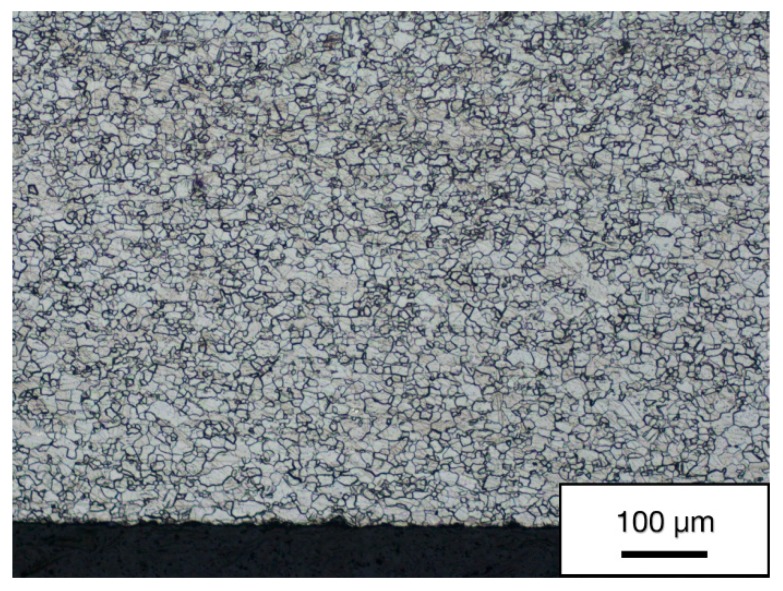
Microstructure in the as-received state in the transverse direction.

Figure 3 is a micrograph of the as-received material which contains recrystallized equiaxed grains with annealing twins. The presence of twins is associated with a low degree of symmetry of the hexagonal lattice [25]. The average as-received grain size was 22 μm.

**Figure 4:**
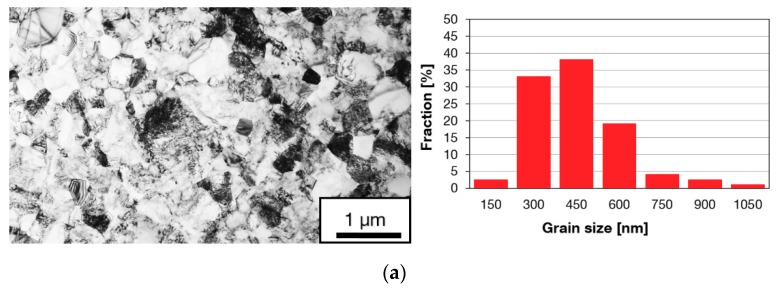

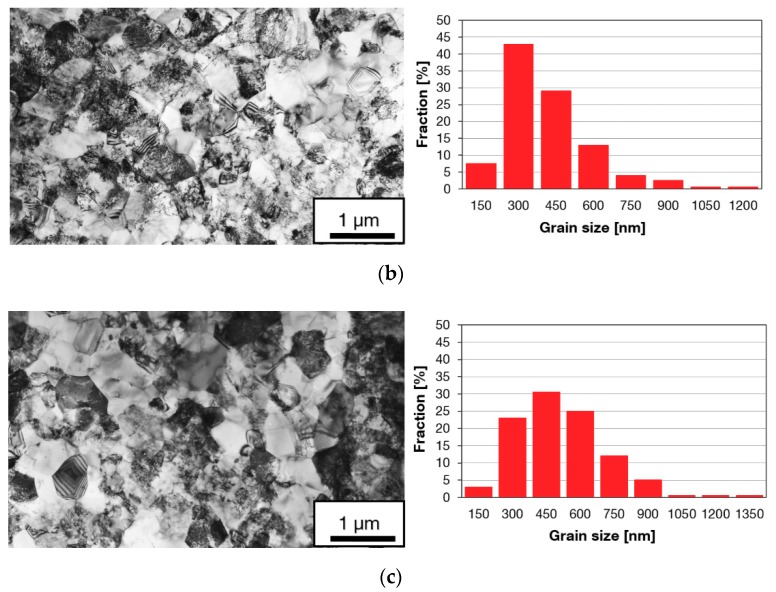
Substructures and grain size histograms in the transverse direction: (**a**) After the first pass; (**b**) after the second pass; (**c**) after the third pass through the Conform SPD machine.

**Figure 5:**
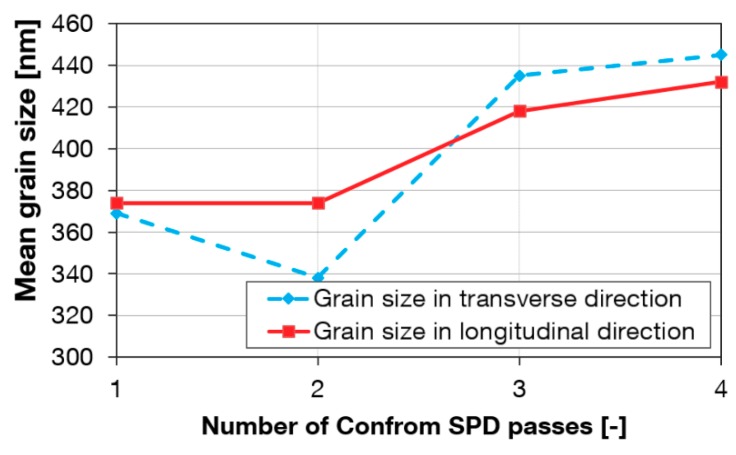
Mean grain size vs. number of passes through the Conform SPD machine.

Figure 4 presents substructures of the material after various numbers of passes through the Conform SPD machine. Each micrograph is accompanied by a histogram of grain size distribution. The most notable refinement occurred during the first pass through the Conform SPD machine. The resultant grain size was approximately 374 nm (Figure 4a). Most grains were polyhedral but some regions contained acicular grains (centres of micrographs in Figure 4). There were some occasional grains whose size exceeded 1 μm, as indicated by the histograms. Dislocation density was very non-uniform. In some regions, there were grains with very low dislocation densities, in others, grains with high dislocation densities. No deformation twins were found, which, at this strain magnitude, is consistent with the findings in [23]. The non-uniform substructure can be seen after the Conform SPD processing, after one or more passes. Subsequent passes did not lead to further refinement of the substructure (Figure 4b,c). The number of grains with a grain size of 700–900 nm increased after the third pass through the Conform SPD machine (Figure 4c). The relationship between the grain size and the number of extrusion passes is plotted in Figure 5 for the transverse and longitudinal directions. In Figure 5, an increase in the grain size can be seen after the third and fourth passes through the Conform SPD machine.

**Figure 6:**
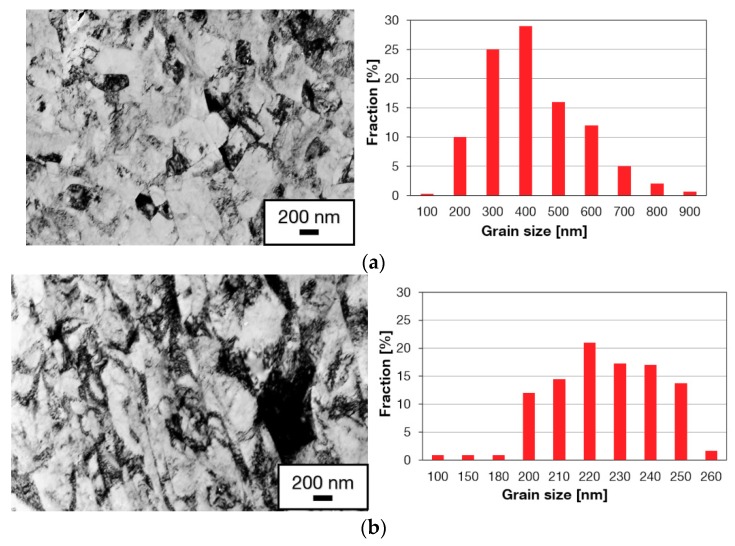
Substructure and grain size histogram after three passes through the Conform SPD and rotary swaging (60% reduction of cross-sectional area): (**a**) in the transverse direction; (**b**) in the longitudinal direction.

**Figure 7:**
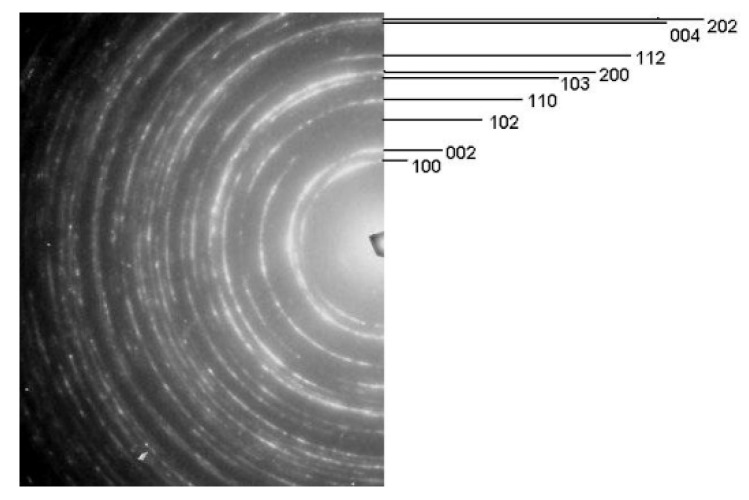
Diffraction spectrum for α-Ti with higher intensities of the diffraction lines for {100} planes.

Figure 6 presents the substructures after three passes through the Conform SPD machine and rotary swaging (true strain 0.9%–60% reduction of the cross-sectional area). Figure 6a shows the substructure in the transverse direction, in which equiaxed polyhedral grains dominate. The mean grain size in the transverse direction was 370 nm. Figure 6b shows the substructure in the longitudinal direction. It contains elongated grains. The related histogram shows the width of the elongated grains. As the diffraction spectra obtained from these samples contain more intensive diffraction lines for the {100} planes than those from the region of polyhedral equiaxed grains, one can assume that the preferential grain orientation is associated with these planes (Figure 7). It is a basal texture which is characteristic of pure titanium after conventional cold forming [26]. Whereas rotary swaging has not led to further refinement, it produced higher dislocation densities, strong texture and elongated grains. These are characteristic features of work hardening [26].

**Figure 8:**
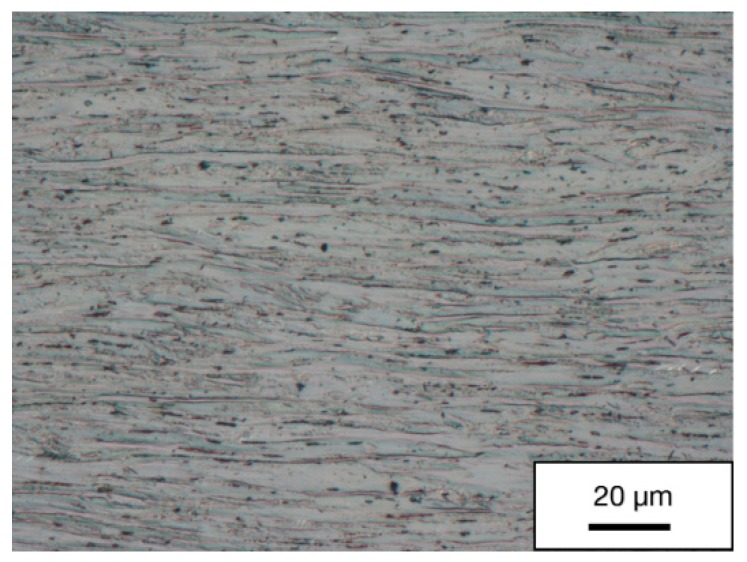
Micrograph of rotary-swaged material in the longitudinal direction after a 90% reduction of cross-sectional area.

To compare, Figure 8 depicts the microstructure of the material after plain rotary swaging (reduction of area −90%) for comparison with the microstructure achieved after processing by combination of Conform SPD and rotary swaging presented in Figure 6b. The purpose was to examine the effect of an added SPD process on the microstructure characteristics. The photograph shows that the initial grains became significantly elongated. The grain size in the longitudinal direction was much larger.

### 3.3. Mechanical Propeties

**Figure 9:**
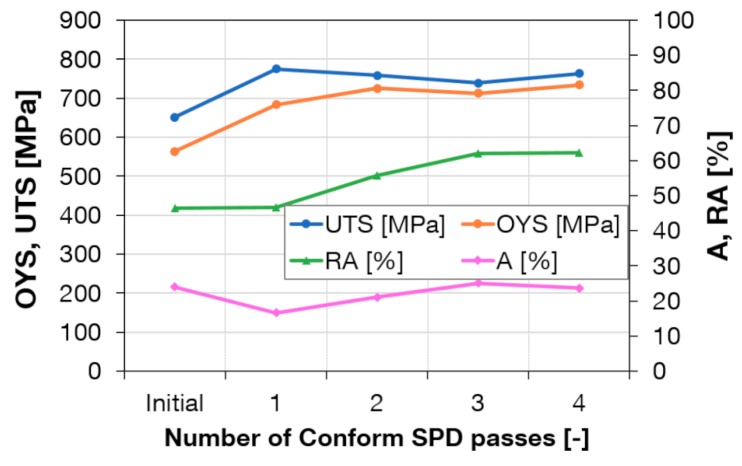
Mechanical properties vs. number of passes through the Conform SPD machine.

Figure 9 is a plot of mechanical properties vs. number of passes through the Conform SPD machine. Work-hardening was most intensive in the first pass. Ultimate strength increased from 651 MPa to 777 MPa. This is in good agreement with the change of grain size values after the first pass according to Hall–Petch strengthening. Further passes did not lead to an increase in strength. Figure 9 indicates that the processing has not reduced elongation. The RA even increased slightly. Hence, Conform SPD processing did not decrease material ductility.

**Figure 10:**
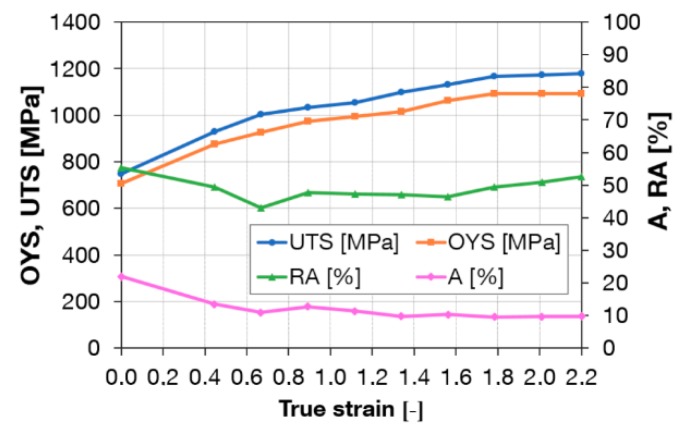
Tensile properties after one pass through the Conform SPD machine and after rotary swaging in relation to true strain.

**Figure 11:**
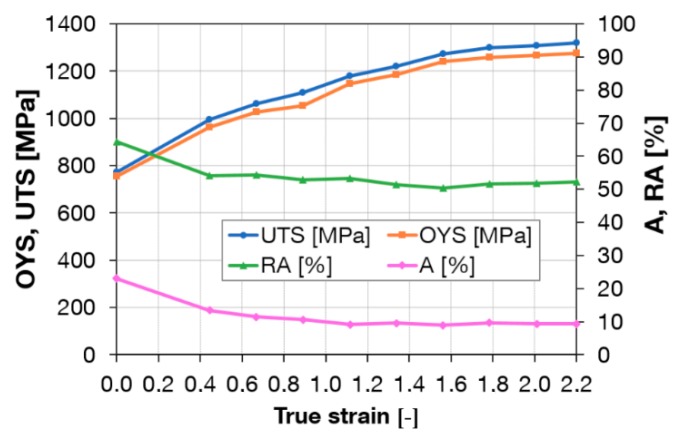
Tensile properties after three passes through the Conform SPD machine and after rotary swaging in relation to true strain.

**Figure 12:**
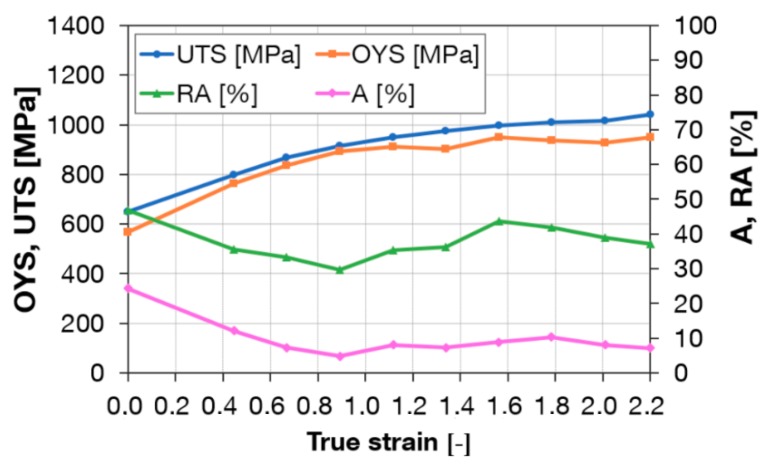
Tensile properties after rotary swaging in relation to true strain.

Workpieces after one and three passes through Conform SPD were rotary-swaged. Rotary swaging is close to conventional forming methods. Figure 10 shows a plot of mechanical properties after a single pass through the Conform SPD machine and rotary swaging. A steep increase in the ultimate strength and yield strength with reduction using rotary swaging can be seen in the graph. After multiple steps using rotary swaging to the true strain of 2.2 (90% reduction), the ultimate strength reached 1180 MPa. By contrast, elongation decreased to 10%. This is consistent with work hardening processes in which the plastic deformation capability of metal is gradually reduced and eventually depleted. Figure 11 shows a plot of mechanical properties after three passes through the Conform SPD machine and rotary swaging. After a true strain of 2.2, the ultimate strength reached 1320 MPa. Elongation and reduction of area remained unchanged when compared to a single pass. After three passes, the increase in ultimate strength was greater than after a single pass. For the sake of comparison, Figure 12 presents the mechanical properties of plain rotary-swaged material. At a true strain of 2.2, the material reached a strength of 1050 MPa, which is approximately 130 MPa less than the material upon a single Conform SPD pass and rotary swaging.

### 3.4. Hardness Measurement

**Figure 13:**
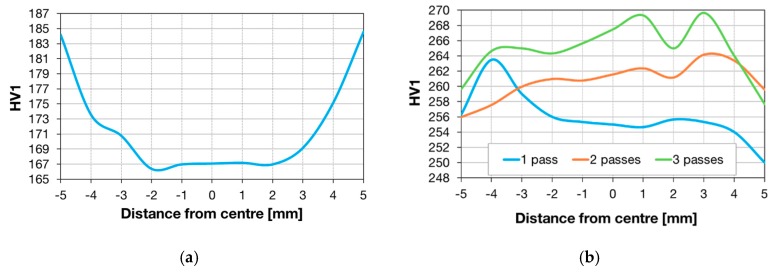
Hardness measurement: (**a**) HV1 hardness profile, as-received condition; (**b**) HV1 hardness profiles vs. number of passes through Conform SPD.

The plots in Figure 13 show the hardness on the transverse cross-section for the as-received and Conform SPD processed conditions. Figure 13a presents the data for the as-received material. The hardness near the surface was higher than in the centre of the wire. Figure 13b shows transverse profiles of hardness for material processed in the Conform SPD machine with various numbers of passes. Generally, the hardness values increase in comparison to the input state of the material. The higher hardness values are more noticeable for the workpiece after three passes. Hardness after Conform SPD processing was lower near the surface.

## 4. Discussion

The metallographic analysis shows a non-uniform substructure which is probably due to variation in the deformation conditions across the workpiece cross-section during the Conform SPD process. Results of numerical modelling appear to confirm this (Figure 2) together with the hardness results over the cross-section (Figure 13b). The grain size with increasing passages levelled out as the fraction of equiaxed grains with lower dislocation density increased. The likely cause lies in the thermodynamic conditions of deformation in the Conform SPD machine. As the process is continuous, it leads to a constant process temperature. It is referred to as an adiabatic state while no heat is exchanged between the workpiece and the tools owing to high process speeds [27,28]. Thermodynamic conditions then preclude further grain refinement. In order to enable further refinement, an intervention would be necessary, possibly in the form of providing a means to dissipate heat. This is less of a problem in the ECAP method which leads to gradual refinement of the substructure. It is probably due to more effective removal of heat through the tool during the cyclic process, as there is a steeper temperature gradient between the workpiece and the tool. It appears that with further passes, the grains merely rotate with respect to one another, as evidenced by [8]. The increase in the grain size after multiple passes through the Conform SPD machine might be due to deformation heat (Figure 2). Deformation heat activates either dynamic or post-dynamic softening processes. The activation temperatures of these processes are depressed by the stored deformation energy, which has a strong impact on recovery process kinetics [11].

The purpose of showing the micrographs after only rotary swaging was to demonstrate the effect of this process on the material. It was demonstrated that the rotary swaging process on its own cannot be considered an SPD process as nano grain size was found for pure titanium.

More Conform SPD passes did not increase the strength values. The grain size data offer reliable evidence of this phenomenon (Figure 5). Mishra et al. attribute this to mutual rotation of equiaxed grains [3]. The process repeats within the sub-grains until the size becomes sufficiently small such that the sub-grains can rotate. Additional deformation causes the sub-grains to rotate into high-angle grain boundaries, typically with an equiaxed shape [1,3]. TEM micrographs show predominantly grains with high-angle boundaries (Figure 4). In this investigation, the Conform SPD processing did not decrease material ductility. Valiev et al. reported similar behaviour in HPT-processed titanium which exhibited a good combination of ductility and strength. According to these authors, the non-equilibrium grain boundaries are high-angle boundaries, i.e., those with large angular misorientation. They have high dislocation density, excess energy and long-range stress fields. Under these conditions, UFG metals are expected to experience grain boundary sliding even at 20 °C [29]. After three passes, the increase in ultimate strength was greater than after a single pass, probably due to the character of the microstructure. The substructure obtained with three passes was more uniform. The fraction of grains with high-angle boundaries and lower dislocation density was larger. This type of substructure appears to exhibit stronger work hardening.

The higher hardness of input material near the surface is due to processing history, as the wire was made by rolling. Rolling leads to greater hardening near the surface [30]. Hardness was higher for the Conform SPD processed wires then for the feedstock material. Although hardness has increased, its values were non-uniform, probably due to non-uniform deformation conditions, as shown by numerical modelling (Figure 2). The lower hardness near the surface after Conform SPD processing was found despite the higher amount of strain (Figure 2c). Similar effects were observed in extruded aluminium sections [31]. One can assume that higher strain in the surface regions (due to friction between the workpiece and the tools) depresses their recrystallization temperature below that of the centre of the workpiece. As a result, the near-surface region is likely to undergo partial recovery. For UFG materials, reduction in recrystallization temperatures was reported in several studies [11,32,33]. 

All in all, ultra-fine to nano-grained titanium Grade 4 could replace conventional titanium Grade 5. In this work, titanium Grade 4 reached ultimate strengths up to 1320 MPa. Also, the advantage of better biocompatibility of pure titanium is an important criterion for dental implant production. At this moment, extensive research on Conform SPD and rotary swaged processed titanium Grade 4 in COMTES FHT Company is continuing. The fatigue properties and biological performance in the human body are being investigated.

## 5. Conclusions

A concept of a continuous process achieving ultrafine to nanocrystalline grains was developed for wires made of commercially pure titanium Grade 4. The Conform SPD process was employed in combination with rotary swaging. After Conform SPD processing, the substructure contained equiaxed polyhedral grains with a mean size of 374 nm. The largest refinement was achieved with the first pass. Subsequent passes did not lead to any further refinement. Subsequent rotary swaging produced higher dislocation densities and elongated the grains in the direction of the material flow. A strong basal texture was produced. Conform SPD processing with a single pass led to increased strengths, at the level of 770 MPa, and no change in elongation. Further passes did not improve the strength characteristics. Additional strengthening was achieved thanks to rotary swaging which led to strengths of up to 1320 MPa. This was accompanied by a decrease in elongation of approximately 10%. These results demonstrate the strong potential of ultrafine to nanocrystalline titanium application for medical implants.

## Figures and Tables

**Table 1:** Chemical composition of the experimental material (weight %).

Element	Fe	O	C	H	N
Titanium Grade 4	0.5	0.4	0.1	0.0125	0.05

## References

[1] Sabirov I., Enikeev N.A., Murashkin M.Y., Valiev R.Z. (2015). Bulk Nanostructured Materials with Multifunctional Properties.

[2] Mishnaevsky L., Levashov E., Valiev R.Z., Segurado J., Sabirov I., Enikeev N., Prokoshkin S., Solov’Yov A.V., Korotitskiy A., Gutmanas E. (2014). Nanostructured titanium-based materials for medical implants: Modeling and development. Mater. Sci. Eng. R Rep..

[3] Mishra A., Kad B.K., Gregori F., Meyers M.A. (2007). Microstructural evolution in copper subjected to severe plastic deformation: Experiments and analysis. Acta Mater..

[4] Gomes C.C., Moreira L.M., Santos V.J.S.V., Ramos A.S., Lyon J.P., Soares C.P., Santos F.V. (2011). Assessment of the genetic risks of a metallic alloy used in medical implants. Genet. Mol. Biol..

[5] Okazaki Y., Gotoh E., Manabe T., Kobayashi K. (2004). Comparison of metal concentrations in rat tibia tissues with various metallic implants. Biomaterials.

[6] Lin C.W., Ju C.P., Chern Lin J.H. (2005). A comparison of the fatigue behavior of cast Ti-7.5Mo with c.p. titanium, Ti-6Al-4V and Ti-13Nb-13Zr alloys. Biomaterials.

[7] Zreiqat H., Valenzuela S.M., Ben Nissan B., Roest R., Knabe C., Radlanski R.J., Renz H., Evans P.J. (2005). The effect of surface chemistry modification of titanium alloy on signalling pathways in human osteoblasts. Biomaterials.

[8] Raab G.I., Valiev R., Gunderov D., Lowe T.C., Misra A., Zhu Y.T. (2009). Long-Length Ultrafine-Grained Titanium Rods Produced by ECAP-Conform. Mater. Sci. Forum.

[9] Zemko M., Kubina T., Dlouhý J., Kover M., Hodek J. (2014). Technological aspects of preparation of nanostructured titanium wire using a CONFORM machine. IOP Conf. Ser. Mater. Sci. Eng..

[10] Palán J., Maleček L., Hodek J., Zemko M., Dzugan J. (2017). Possibilities of biocompatible material production using conform SPD technology. Arch. Mater. Sci. Eng..

[11] Kubina T., Dlouhý J., Kövér M., Hodek J. (2014). Study of Thermal Stability of Ultra Fine-Grained Commercially Pure Titanium Wire Prepared in Conform Equipment. Mater. Sci. Forum.

[12] Li B., Li C.H., Yao X.J., Song B.Y. (2011). Effects of Continuous Extrusion on Microstructure Evolution and Property Characteristics of Brass Alloy. Adv. Mater. Res..

[13] He Y.L., Gao F., Song B.Y., Fu R., Wu G.M., Li J., Jiang L. (2012). Grain Refinement of Magnesium Alloys by CONFORM: A Continuous Severe Plastic Deformation Route?. Mater. Sci. Forum.

[14] Etherington C. (1973). Conform—A New Concept for the Continuous Extrusion Forming of Metals. J. Manuf. Sci. Eng. Trans. ASME.

[15] Palán J., Procházka R., Džugan J., Nacházel J., Duchek M., Gergely N., Minárik P., Horvát K. (2018). Comprehensive Evaluation of the Properties of Ultrafine to Nanocrystalline Grade 2 Titanium Wires. Materials.

[16] Valiev R.Z., Langdon T.G. (2006). Principles of equal-channel angular pressing as a processing tool for grain refinement. Prog. Mater. Sci..

[17] Xu C., Schroeder S., Berbon P.B., Langdon T.G. (2010). Principles of ECAP-Conform as a continuous process for achieving grain refinement: Application to an aluminum alloy. Acta Mater..

[18] Zhao X., Yang X., Liu X., Wang X., Langdon T.G. (2010). The processing of pure titanium through multiple passes of ECAP at room temperature. Mater. Sci. Eng. A.

[19] Krystian M., Huber D., Horky J. (2017). Equal channel angular pressing (ECAP) and forging of commercially pure titanium (CP-Ti). AIP Conf. Proc..

[20] Semenova I.P., Valiev R.Z., Yakushina E.B., Salimgareeva G.H., Lowe T.C. (2008). Strength and fatigue properties enhancement in ultrafine-grained Ti produced by severe plastic deformation. J. Mater. Sci..

[21] Semenova I.P., Polyakov A.V., Raab G.I., Lowe T.C., Valiev R.Z. (2012). Enhanced fatigue properties of ultrafine-grained Ti rods processed by ECAP-Conform. J. Mater. Sci..

[22] Wu H., Jiang J., Liu H., Sun J., Gu Y., Tang R., Zhao X., Ma A. (2017). Fabrication of an Ultra-Fine Grained Pure Titanium with High Strength and Good Ductility via ECAP plus Cold Rolling. Metals (Basel).

[23] Gunderov D.V., Polyakov A.V., Semenova I.P., Raab G.I., Churakova A.A., Gimaltdinova E.I., Sabirov I., Segurado J., Sitdikov V.D., Alexandrov I.V. (2013). Evolution of microstructure, macrotexture and mechanical properties of commercially pure Ti during ECAP-conform processing and drawing. Mater. Sci. Eng. A.

[24] Hodek J., Zemko M. (2013). FEM model of continuous extrusion of titanium in deform software. Tanger Ltd. Plzeň Czech Repub..

[25] Hatherly F.J.H., Hatherly M. (2004). Recrystallization and Related Annealing Phenomena.

[26] Nourbakhsh S., O’Brien T.D. (1988). Texture formation and transition in Cold-rolled titanium. Mater. Sci. Eng..

[27] Thomas B.M., Derguti F., Jackson M. (2017). Continuous extrusion of a commercially pure titanium powder via the Conform process. Mater. Sci. Technol. (UK).

[28] Thomas B.M. (2015). Continuous Extrusion of Commercially Pure Titanium Powder.

[29] Valiev R.Z., Alexandrov I.V. (2000). Nanostructured materials from severe plastic deformation. Prog. Mater. Sci..

[30] Jansson B., Rolfson M., Thuvander A., Melander A., Wullimann C. (2014). Calculation of microstructure and hardness of hot rolled steel bars. Mater. Sci. Technol..

[31] Eivani A.R., Zhou J., Duszczyk J. (2016). Mechanism of the formation of peripheral coarse grain structure in hot extrusion of Al-4.5Zn-1Mg. Philos. Mag..

[32] Kubina T., Dlouhý J., Köver M., Dománková M., Hodek J. (2015). Preparation and thermal stability of ultra-fine and nano-grained commercially pure titanium wires using conform equipment. Mater. Tehnol..

[33] Palán J., Procházka R., Zemko M. (2017). The microstructure and mechanical properties evaluation of UFG Titanium Grade 4 in relation to the technological aspects of the CONFORM SPD process. Procedia Eng..

